# Dataset of allele, genotype and haplotype frequencies of five polymorphisms CDKN2B-AS1 gene in Russian patients with primary open-angle glaucoma

**DOI:** 10.1016/j.dib.2020.105722

**Published:** 2020-05-18

**Authors:** Natalya Eliseeva, Irina Ponomarenko, Evgeny Reshetnikov, Alexey Polonikov, Irina Batlutskaya, Inna Aristova, Anna Elykova, Natalya Rudykh, Mikhail Churnosov

**Affiliations:** a Department of Medical Biological Disciplines, Belgorod State University, 308015 Belgorod, Russia; b Department of Biology, Medical Genetics and Ecology, Kursk State Medical University, 305041 Kursk, Russia

**Keywords:** Single nucleotide polymorphism, Primary open-angle glaucoma, Female, Male, *CDKN2B-AS* gene

## Abstract

Data on the allele, genotype and haplotype frequencies of the five single nucleotide polymorphisms (SNPs) such as rs1063192, rs7865618, rs2157719, rs944800 and rs4977756 of the *CDKN2B-AS* gene in Russian patients with primary open-angle glaucoma (POAG) are provided. These SNPs are found to be associated with the risk of POAG by genome-wide association studies (GWAS). The frequencies of alleles, genotypes and haplotypes of *CDKN2B-AS* gene were present separately for entire group of patients, females and males, and may be used as reference data of Russian population.

**Specifications table**

**Table:** 

Subject	*Biology*
Specific subject area	*Genetics*
Type of data	*Table*
How data were acquired	*MALDI/TOF mass spectrometry using Sequenom MassARRAY 4.0 platform (Agena Bioscience™)*
Data format	*Raw and analyzed data*
Parameters for data collection	*About 5 ml of whole blood was collected from each study subject into a plastic vial (Vacutainer®) with 0.5M EDTA (рН=8.0). Genomic DNA was isolated using standard method of phenol-chloroform extraction and purification. DNA samples of good quality (concentration 10-15 ng/mL, purity А260/А280=1.7-2.0) were included for genotyping. About 5% of blind replicate samples were included for quality control of genotyping, and the repeatability test resulted in a 100% concordance rate.*
Description of data collection	*The quality of isolated DNA was assessed by Nanodrop-2000 spectrophotometer. DNA samples were genotyped using Sequenom MassARRAY® iPLEX platform using a method of MALDI-TOF (matrix-assisted laser desorption/ ionization time-of-flight) mass spectrometry. Assay Design Suite 1.0 was used to design a multiplex genotyping assay (http:// agenabio.com/assay-design-suite-10-software).*
Data source location	*Belgorod, Russia*
Data accessibility	*The data is available with this article*

## Value of the data

• The frequencies of alleles, genotypes and haplotypes for five SNPs such as rs1063192, rs7865618, rs2157719, rs944800 and rs4977756 of the *CDKN2B-AS1* gene are presented separately for entire sample, males and females with POAG from Russian population.
• The data on the allele, genotype and haplotypes frequencies represent a resource for conducting meta-analyses of genetic studies on POAG.
• Allele, genotype and haplotype frequencies of the *CDKN2B-AS1* gene polymorphisms and linkage disequilibrium values can be used as reference data for further population and genetic association studies of common diseases.

## 1 Data Description

The dataset represents the raw data (supplementary Table), frequencies of alleles, genotypes (Table 1) and haplotypes (Table 2) for five SNPs (rs1063192, rs7865618, rs2157719, rs944800 and rs4977756) of the *CDKN2B-AS* gene in Russian patients with POAG. These SNPs are found to be associated with the risk of POAG in previously published GWAS (Table 3) [1], [2], [3], [4], [5], [6], [7], [8], [9], [10]. These SNPs possess the regulatory potential (Table 4), as demonstrated by several eQTLs (Table 5) and splicing QTLs (Table 6). The frequencies of alleles, genotypes and haplotypes for the SNPs are provided separately for three groups: entire sample, females and males. No significant differences in the allele, genotype and haplotype frequencies were found between the males and females groups.

**Table 1:** The frequencies of alleles and genotypes for SNPs rs1063192, rs7865618, rs2157719, rs944800 and rs4977756 *CDKN2B-AS1* gene in Russian patients with POAG.

SNP genotype or allele	All (n=536)		Female (n=290)		Male (n=246)	
	n	frequency	n	frequency	n	frequency
rs1063192						
GG	104	0.1940	59	0.2034	45	0.1829
AG	256	0.4776	134	0.4621	122	0.4959
AA	176	0.3284	97	0.3345	79	0.3212
G	464	0.4328	252	0.4345	212	0.4309
A	608	0.5672	328	0.5655	280	0.5691
rs7865618						
GG	94	0.1753	52	0.1793	42	0.1707
AG	263	0.4907	139	0.4793	124	0.5041
AA	179	0.3340	99	0.3414	80	0.3252
G	451	0.4207	243	0.4190	208	0.4228
A	621	0.5793	337	0.5810	284	0.5772
rs2157719						
GG	85	0.1585	45	0.1552	40	0.1626
AG	249	0.4646	135	0.4655	114	0.4634
AA	202	0.3769	110	0.3793	92	0.3740
G	419	0.3909	225	0.3879	11	0.3943
A	653	0.6091	355	0.6121	215	0.6057
rs944800						
AA	64	0.1194	32	0.1103	32	0.1301
GA	241	0.4496	130	0.4483	111	0.4512
GG	231	0.4310	128	0.4414	103	0.4187
A	369	0.3442	194	0.3345	175	0.3557
G	703	0.6558	386	0.6655	317	0.6443
rs4977756						
GG	100	0.1866	61	0.2104	39	0.1585
AG	286	0.5336	154	0.5310	132	0.5366
AA	150	0.2798	75	0.2586	75	0.3049
G	486	0.4534	276	0.4759	210	0.4268
A	586	0.5466	304	0.5241	282	0.5732

**Table 2:** The frequencies of haplotypes for SNPs rs1063192, rs7865618, rs2157719, rs944800 and rs4977756 *CDKN2B-AS1* gene in Russian patients with POAG.

Haplotype (rs1063192-rs7865618-rs2157719-rs944800-rs4977756)	All (n=536), frequency	Female (n=290), frequency	Male (n=246), frequency
GGGAG	0.2221	0.2197	0.2304
AGGAG	0.0127	0.0125	0.0130
GAGAG	0.0220	0.0189	0.0272
AAGAG	0.0101	0.0137	0.0068
GGAAG	0.0120	0.0185	0.0054
GGGGG	0.0505	0.0628	0.0434
GGAGG	0.0373	0.0361	0.0397
AAAGG	0.0713	0.0859	0.0641
GGGAA	0.0262	0.0211	0.0359
AAAAA	0.0299	0.0265	0.0377
AGGGA	0.0133	0.0178	0.0096
AAGGA	0.0141	0.0155	0.0136
GGAGA	0.0154	0.0117	0.0208
AGAGA	0.0191	0.0189	0.0198
GAAGA	0.0318	0.0424	0.0217
AAAGA	0.3890	0.3779	0.4110

**Table 3:** The literature data about associations of the studied polymorphisms *CDKN2B-AS1* gene with POAG and optic disc characteristics (GWAS data).

SNP	Position (hg38)	Phenotype	Association (significance)(associated allele)	Reference
rs1063192	22003368	POAG Vertical cup-disc ratio	OR= 0.79 (p=5 × 10^−11^) (T) β = -0.01 mm^2^ (p=4 × 10^−15^) (G)	[1] [2]
rs7865618	22031006	POAG Vertical cup-disc ratio Optic cup area	OR= 1.78 (p=9 × 10^−11^) (A) β = -0.013 unit (p=5 × 10^−24^) (G) β = -0.023 unit (p=1 × 10^−21^) (G)	[3] [4] [5]
rs2157719	22033367	POAG POAG Vertical cup-disc ratio	OR= 1.45 (p=2 × 10^−18^) OR= 1.41 (p= 3 × 10^−33^) β = -0.013 unit (p=4 × 10^−35^)	[6] [7] [8]
rs944800	22050899	POAG	OR= 1.33 (p=4 × 10^−14^) (G)	[9]
rs4977756	22068653	POAG	OR= 1.48 (p= 7 × 10^−30^) (A)	[10]

**Table 4:** Regulatory effects of the 5 SNPs of the CDKN2B-AS1 gene (HaploReg, v4.1, update 05.11.2015) (https://pubs.broadinstitute.org/mammals/haploreg/haploreg.ph

chr	pos (hg38)	variant	Ref	Alt	AFR	AMR	ASN	EUR	SiPhy	Promoter	Enhancer	DNAse	Proteins	Motifs	NHGRI/EBI	GRASP QTL	Selected eQTL	GENCODE	dbSNP
					freq	freq	freq	freq	cons	histone marks	histone marks		bound	changed	GWAS hits	hits	hits	genes	func annot
9	22003368	rs1063192	G	A	0.99	0.79	0.82	0.57				SKIN		AIRE,GATA,Tgif1	2 hits	3 hits		CDKN2B	3′-UTR
9	22031006	rs7865618	G	A	0.99	0.8	0.9	0.58			BLD, SKIN				3 hits	4 hits		RP11-145E5.5	intronic
9	22033367	rs2157719	C	T	0.99	0.8	0.9	0.58			BRST, SKIN	6 tissues		Pou2f2,Zfp187	3 hits	4 hits		CDKN2B-AS1	intronic
9	22050899	rs944800	A	G	0.99	0.86	0.9	0.68			12 tissues	4 tissues		GATA				CDKN2B-AS1	intronic
9	22068653	rs4977756	G	A	0.67	0.78	0.79	0.6				BRN			4 hits	2 hits		CDKN2B-AS1	intronic

**Table 5:** The cis-eQTL values of the 4 SNPs of the *CDKN2B-AS1* gene. (according to Genotype-Tissue Expression (GTEx) (http://www.gtexportal.org/)).

SNP	Gene expression	Reference allele	Alternative allele	Effect Size (β)	P-Value	Tissue
rs1063192	*CDKN2A*	G	A	0.33	0.000031	Brain - Cortex
	*CDKN2B*	G	A	-0.14	0.000051	Muscle - Skeletal
rs7865618	*CDKN2A*	G	A	0.33	0.000045	Brain - Cortex
	*CDKN2B*	G	A	-0.14	0.000085	Muscle - Skeletal
rs2157719	*CDKN2A*	C	T	0.33	0.000045	Brain - Cortex
	*CDKN2B*	C	T	-0.14	0.00009	Muscle - Skeletal
rs944800	*CDKN2B-AS1*	A	G	0.26	0.0000026	Cells - Transformed fibroblasts

**Table 6:** The sQTL values of the 5 SNPs of the *CDKN2B-AS1* gene (according to Genotype-Tissue Expression (GTEx) (http://www.gtexportal.org/)).

SNP	Gene Symbol	Reference allele	Alternative allele	Intron Id	Effect Size (β)	P-Value	Tissue
rs1063192	*CDKN2B-AS1*	G	A	21995161:22046751:clu_55270	0.47	7.9e-9	Pituitary
	*RP11-149I2.4*	G	A	21995161:22046751:clu_55270	0.47	7.9e-9	Pituitary
rs7865618	*CDKN2B-AS1*	G	A	21995161:22046751:clu_55270	0.47	1.2e-8	Pituitary
	*RP11-149I2.4*	G	A	21995161:22046751:clu_55270	0.47	1.2e-8	Pituitary
rs2157719	*CDKN2B-AS1*	C	T	21995161:22046751:clu_55270	0.47	1.3e-8	Pituitary
	*RP11-149I2.4*	C	T	21995161:22046751:clu_55270	0.47	1.3e-8	Pituitary
rs944800	*CDKN2B-AS1*	A	G	21995161:22046751:clu_55270	0.4	0.000004	Pituitary
	*RP11-149I2.4*	A	G	21995161:22046751:clu_55270	0.4	0.000004	Pituitary
rs4977756	*CDKN2B-AS1*	G	A	21995161:22046751:clu_55270	0.43	9.3e-8	Pituitary
	*RP11-149I2.4*	G	A	21995161:22046751:clu_55270	0.43	9.3e-8	Pituitary

## 2 Experimental Design, Materials, and Methods

### 2.1 Study subjects

A study sample was comprised of 536 patients with POAG, including 290 females and 246 males. The study participants were examined at the Division of Eye Microsurgery of Saint Joasaph's Belgorod Regional Clinical Hospital. The patients with POAG were unrelated Russians, residents of the Central Russia [11]. The subjects were enrolled for the study according to criteria described elsewhere [12]. All study participants signed a written informed consent in accordance with the principles of the Helsinki Declaration.

### 2.2 DNA analysis

Whole blood sample (5 ml) from each participant was drawn by a certified nurse into a plastic vial (Vacutainer®) with 0.5M EDTA (рН=8.0). Total DNA was isolated from buffy coat using standard phenol-chloroform extraction method [13]. DNA quality was assessed by Nanodrop-2000 spectrophotometer (Thermo Scientific, Inc.). DNA samples of good quality (concentration 10-15 ng/mL, purity А260/А280=1.7-2.0) were included for genotyping.

Five SNPs such as rs1063192, rs7865618, rs2157719, rs944800 and rs4977756 of the *CDKN2B-AS* gene were selected for the study according to the following criteria [14,15]: 1) SNP showed an association with POAG by GWAS, 2) SNP possesses the regulatory potential, 3) SNP has eQTLs and/or sQTLs, 4) minor allele frequency, MAF > 5%.

All selected SNPs were associated with POAG in previously published GWAS (Table 3). These SNPs have the regulatory potential (Table 4), eQTLs (Table 5) and sQTLs (Table 6), as assessed by the HaploReg v4.1 (https://pubs.broadinstitute.org/mammals/haploreg/haploreg.php) and GTExportal recourses (http://www.gtexportal.org).

DNA samples were genotyped using the MALDI‐TOF mass spectrometry iPLEX platform (Agena Bioscience Inc, San Diego, CA). Concentration of DNA varied from 10 to 15 ng/mL. Assay Design Suite 1.0 (http:// agenabio.com/assay-design-suite-10-software) was used to design a multiplex genotyping assay. About 5% of blind replicate samples were included for quality control of genotyping, and the repeatability test resulted in a 100% concordance rate.

### 2.3 Statistical analysis

Allele frequencies were estimated by the gene counting method, and the chi-square test was applied to identify significant departures from Hardy–Weinberg equilibrium (HWE). Differences in allele, genotype and haplotype frequencies between the study groups (females and males) were analyzed by the Kruskal-Wallis test. The haplotypes for the SNPs of the *CDKN2B-AS1* gene were constructed using an algorithm implemented in the PLINK software, v. 2.050 [16] (http://zzz.bwh.harvard.edu/plink/).

## Declaration of Competing Interest

The authors declare that they have no competing financial interests or personal relationships which have, or could be perceived to have, influenced the work reported in this article.

## Appendix Supplementary materials

Image, application 1

Image, application 2
